# Characterization of protein tyrosine phosphatase H1 knockout mice in animal models of local and systemic inflammation

**DOI:** 10.1186/1476-9255-7-16

**Published:** 2010-03-30

**Authors:** Claudia Patrignani, David T Lafont, Valeria Muzio, Béatrice Gréco, Rob Hooft van Huijsduijnen, Paola F Zaratin

**Affiliations:** 1 MerckSerono Ivrea, In vivo Pharmacology Department, via ribes 5, 10010 Colleretto G. (TO) Italy; 2 University of Eastern Piedmont, Department of Medical Sciences, via solaroli 17, 28100 Novara, Italy; 3 Wellcome Trust Sanger Institute, Team 109, Hinxton, CB1 1SA Cambridge, UK; 4 Advanced Accelerator Applications, Research & Development Pharmacology Department, via Ribes 5, 10010 Colleretto Giacosa (TO), Italy; 5 Merck Serono International S.A., Innovation and Partnerships Department, 9 chemin des mines, 1211 Geneva, Switzerland; 6 Merck Serono International S.A., Molecular Neurobiology MS Department, Geneva Research Center, 9 Chemin de Mines, 1202 Geneva, Switzerland; 7 Scientific Research Department, Associazione Italiana Sclerosi Multipla Onlus, Via Operai 40, 16149 Genova, Italy

## Abstract

**Background:**

PTPH1 is a protein tyrosine phosphatase expressed in T cells but its effect on immune response is still controversial. PTPH1 dephosphorylates TCRzeta *in vitro*, inhibiting the downstream inflammatory signaling pathway, however no immunological phenotype has been detected in primary T cells derived from PTPH1-KO mice. The aim of the present study is to characterize PTPH1 phenotype in two *in vivo *inflammatory models and to give insights in possible PTPH1 functions in cytokine release.

**Methods:**

We challenged PTPH1-KO mice with two potent immunomodulatory molecules, carrageenan and LPS, in order to determine PTPH1 possible role in inflammatory response *in vivo*. Cytokine release, inflammatory pain and gene expression were investigated in challenged PTPH1-WT and KO mice.

**Results:**

The present study shows that carrageenan induces a trend of slightly increased spontaneous pain sensitivity in PTPH1-KO mice compared to WT (wild-type) littermates, but no differences in cytokine release, induced pain perception and cellular infiltration have been detected between the two genotypes in this mouse model. On the other hand, LPS-induced TNFα, MCP-1 and IL10 release was significantly reduced in PTPH1-KO plasma compared to WTs 30 and 60 minutes post challenge. No cytokine release modulation was detectable 180 minutes post LPS challenge.

**Conclusion:**

In conclusion, the present study points out a slight potential role for PTPH1 in spontaneous pain sensitivity and it indicates that this phosphatase might play a role in the positive regulation of the LPS-induced cytokines release *in vivo*, in contrast to previous reports indicating PTPH1 as potential negative regulator of immune response.

## Background

Innate immunity is the early and relatively nonspecific response to invading pathogens, activated via the Toll-like and T-cell receptors, on antigen presenting cells and on T cells, respectively [1,2]. The intensity and duration of the immune response is under stringent regulation. Tyrosine phosphorylation is a central mechanism in the control of key signaling proteins involved in innate immunity. The role of protein tyrosine kinases (PTKs) has been widely studied but less is known on the protein tyrosine phosphatases (PTPs) responsible for immunoregulation [3].

PTP action on immune response can be either positive or negative, promoting or inhibiting the immune system. SRC homology 2 (SH2)-containing tyrosine phosphatase-2 (SHP-2) has a controversial effect on lymphocyte signaling. Qu and colleagues demonstrated that SHP-2 is essential for erythroid and myeloid cell differentiation [4], and a missense mutation in the ptpn11 gene (encoding for SHP-2 protein) is associated with various forms of leukemia [5]. SHP-2 may also have an inhibitory role on the activation of T and B lymphocytes [6]; SHP-2 can hamper the TRIF (TIR-domain-containing adapter-inducing interferon-β) adaptor protein-dependent TLR4 and TLR3 signal transduction with a consequent block of the pro-inflammatory cytokine production [7]. Another negative regulator of hematopoietic cell development and function is SHP-1 (SRC homology 2 (SH2)-containing tyrosine phosphatase 1), that is mainly expressed in hematopoietic and lymphoid cells [8]. Lymphocyte specific phosphatase, (LYP) and its mouse orthologue PEP (PTP enriched in proline, glutamic acid, serine, and threonine sequences) are predominantly expressed in leukocytes and act as potent negative regulators of the TCR signaling pathway [9]. A specific missense mutation in the LYP encoding gene, ptpn22, has been associated in a highly reproducible manner with autoimmune disease, as type1 diabetes [10] and rheumatoid arthritis [11]. Another PTP involved in the immune processes is PTPMEG, a cytosolic phosphatase expressed in the thymus that is able to dephosphorylates TCRζ ITAMs *in vitro*. Trapping mutant experiments show that PTPMEG inactivation leads to increased activation of the NF-kB pathway [12]. However PTPMEG deletion *in vivo *does not induce TCRζ ITAMs dephosphorylation, and PTPMEG-KO mice do not show obviously altered immune responses [12].

The present study is focused on PTPH1 (also known as PTPN3), a cytosolic PTP that has been proposed to inhibit TCR signaling. PTPH1 overexpression in Jurkat T cells reduces indirectly the TCR-induced serine phosphorylation of Mek, Erk, Jnk and AP-1 leading to a decreased IL-2 gene activation [13]. The indirect effect of PTPH1 could be mediated by the dephosphorylation of one or several signaling components upstream of Mek and Jnk, such as the TCR-associated protein tyrosine kinases (PTK) or their immediate targets. Further studies will be needed to identify the direct substrate for PTPH1. It has been also demonstrated that the FERM (band 4.1, ezrin, radixin, moesin) domain of PTPH1 is necessary for the inhibition of Mek, Erk, Jnk and AP-1 and also for localization of the phosphatase on the plasma membrane of Jurkat T cells [14]. These studies corroborate the hypothesis of a possible role for PTPH1 as negative regulator in TCR signaling. Indeed, biochemical approaches and substrate trapping experiments identify PTPH1, together with SHP-1, as the phosphatases able to interact and to dephosphorylate TCRζ *in vitro *[15]. A comparatively recent *ex vivo *study on PTPH1-KO primary T cells failed to show any significant role of this phosphatase in T cell development and activation, thus excluding a possible function for PTPH1 in the negative regulation of TCR signaling [16]. This discrepancy between *in vitro *and *ex vivo *data has been explained by a possible redundancy effect of PTPMEG, that belongs to the same family protein of PTPH1. As already mentioned, PTPMEG is able to dephosphorylate the TCR ITAMs and to regulate NF-κB [12]. Despite the similarity in protein structure between PTPMEG and PTPH1, no evidence can support the hypothesis of PTPH1 affecting NF-κB pathway. However, the double PTPH1-PTPMEG KO mouse line fails to show a T cell phenotype, indicating that PTPMEG does not compensate for the lack of PTPH1 action in primary T cells [17].

In the present study, we examined the contribution of PTPH1 to the regulation of inflammatory responses in mice with a targeted deletion of PTPH1 gene expression. PTPH1-KO and WT mice were treated with two potent immunomodulatory molecules, carrageenan (CARR) and lipopolysaccharide (LPS). Nociceptive perception and cytokine expression and release have been investigated in these two models of local (carrageenan) and systemic (lipopolysaccharide) inflammation.

## Methods

### Animals

PTPH1-KO mice were generated as described in detail elsewhere [18]. The experiments were performed on adult female mice PTPH1-WT and KO individually housed in top filter cages with free access to food and water, under controlled temperature (21 ± 2°C), and relative humidity (55 ± 10%), on a 12:12 h light-dark cycle. Protection of animals used in the experiment was in accordance with Directive 86/609/EEC, enforced by the Italian D.L. No. 116 of January 27, 1992. Physical facilities and equipment for accommodation and care of animals were in accordance with the provisions of EEC Council Directive 86/609. Animals were allowed to acclimate for 1 week before the beginning of the experiments. All behavioral tests were performed during the light phase and animals were allowed 1-hour habituation to the test room, if different from the holding room, before testing. Testing sequence was randomized between KO and WT animals, and all apparatus were thoroughly cleaned between two consecutive test sections.

### Cytometric beads Array (CBA)

At the end of both inflammatory models, a panel of cytokines was analyzed in blood. At sacrifice whole blood was collected from the heart of the animals and plasma was obtained by centrifugation. 25 μl of plasma were used to quantify the levels of the circulating inflammatory cytokines TNFα, MCP-1, IL-6, IL-10, IFN-γ, IL-12p70 using a mouse inflammation cytometric beads array kit (BD Bioscience), according to the manufacturer's instructions. Data were acquired with a FACSCalibur flow cytometer and analyzed with BD CBA Software (BD Bioscience).

### Carrageenan-induced inflammation

Female PTPH1-KO and WT mice (3 months old) were tested for inflammation-induced edema and hyperalgesia/allodynia. On the test day, n = 7-8 animals per genotype were injected subcutaneously in the right hind paw plantar surface with 30 μL of a solution of 2% carrageenan λ (Sigma, Germany) freshly prepared in saline. 30 μL of saline were injected as control in the controlateral paw. Animals were tested at automated Von Frey and Hargreaves apparatus, to evaluate respectively tactile allodynia and thermal hyperalgesia at 1, 3, 5 and 24 hours after carrageenan/saline injection, followed by paw thickness measurement, using a precision caliper (Mitutoyo, Japan). Mice underwent also to a Catwalk analysis at the same time points. Mice were sacrificed by an intraperitoneal (ip) overdose of thiopental and paws were removed for histological evaluation.

#### Hargreaves' plantar test

Thermal hyper/hypoalgesia was assessed by Hargreaves' plantar apparatus (Plantar test, Ugo Basile, Italy) [19]. The test was performed at 1, 3, 5 and 24 hours after 2% carrageenan injection. Animals were accustomed to the apparatus for 1 hour for 2 days preceding the test. On the test day, animals were individually placed in a clear acrylic box on a glass platform and a removable infrared generator (radiant heat 137 mW/cm^2^/s) was placed underneath the animal's hind paw. The apparatus automatically detected the withdrawal of the paw. Latency of each paw withdrawal was recorded and mean values of left and right paws were used as reaction index for the individual animal. A cut-off of 25 seconds was used to avoid tissue damage in case of absence of response.

#### Automated Von Frey test

Mechanical allodynia was assessed by a Dynamic Plantar Aesthesiometer (Ugo Basile, Italy). The test was performed immediately after the Hargreaves's test. Animals were accustomed to the apparatus for 1 hour, for 2 days proceeding the test day. On the test day, mice were individually placed in a clear acrylic box with a grid floor. A blunted probe was placed under the plantar surface of one hind paw and automatically exerted a constantly increasing force to the plantar surface (from 0 up to 5 grams over 20 s). Force applied (g) at the retraction reflex was automatically recorded. Each hind paw was tested 3 times and mean values used as individual parameter for group statistic.

#### CatWalk

Spontaneous pain was assessed using the CatWalk™ (Noldus Information Technology) gait analysis method [20,21]. Briefly, light from a fluorescent tube was sent through a glass plate. Light rays were completely reflected internally. As soon as the paw of the mouse was in contact with the glass surface, light was reflected downwards. It resulted in a sharp image of a bright paw print. The whole run was recorded by a camera placed under the glass plate.

In the present study, the following parameters related to single paw were analyzed:

• *Duty cycle *(expressed in %): the duty cycle represents stance duration as a percentage of step cycle duration. It is calculated according to the formula: stand duration/(stand + swing phases duration) × 100, where the stand phase is indicated as the time of contact (in seconds) of one paw with the glass plate in a single step cycle and the swing phase is indicated as seconds of non-contact with the plate during a step cycle. The duty cycle parameter is highly correlated with the Von Frey thresholds [22] and it is used to assess pain-related spontaneous behavior in the carrageenan-induced knee joint arthritis [23].

• *Print area *(expressed in mm^2^): this parameter describes the surface area of the complete paw print during the stance phase.

#### Histological Analysis

At sacrifice paws were collected and placed in 4% formalin. Paws were then incubated for 10-15 days in Shandon TBD2 decalcifier (Thermo-Scientific) and subsequently cut in 7 μm thick slices by a microtome. After mounting, the slides were let overnight at 37°C, dehydrated and stained with hematoxylin and eosin in a multiple steps procedure. Histological evaluation was observed by microscopy and described by an operator blind to the genotypes.

### LPS-induced inflammation

PTPH1-WT and KO female mice (n = 3-6, 2 months old) received an ip injection of 1 mg/kg of LPS (Escherichia coli 0127:B8, batch 032K4099, L3880, Sigma) and randomized groups of mice were sacrificed by an ip overdose of thiopental at 30, 60 and 180 minutes after LPS injection. The test was performed in three sessions with equivalent group representation.

#### RTPCR on white cells

At the designed time points, blood was processed for RT-PCR on white cells as follows. Red blood cells were lysed from whole blood with BD PharM Lyse™ lysing solution (BD Biosciences/BD Pharmingen) and whole white cells were washed in PBS. RNA from whole white cells was extracted using TriZol (Invitrogen). 200 ng of total RNA were used to perform the RT-PCR reaction (SuperScript II RT kit, Invitrogen). The qPCR experiment was carried out using the Taqman Universal PCR master mix (Applied Biosystems) on the following cytokine genes: ccl2 (#Mm00441242_m1, Applied Biosystems), IL1b (#Mm01336189_m1, Applied Biosystems), IL12a (#Mm00434169_m1, Applied Biosystems), IL6 (#Mm00446190_m1, Applied Biosystems), TNF (#Mm00443258_m1, Applied Biosystems). The comparative Ct method [24] was used for data analysis, where:

and (delta)Ct_sample _is the Ct value for any sample normalized to the endogenous housekeeping gene (beta-2-microglobin, Applied Biosystems) and (delta)Ct_reference _is the Ct value for matched PTPH1-WT vehicle treated value, also normalized to the endogenous housekeeping gene.

### Statistical analysis

Statistical comparisons were performed by Two-way Anova followed by T-test and Bonferroni's post-hoc analysis (p < 0.05) at each time points. Results are expressed as mean ± SEM.

## Results

### Carrageenan (CARR)-induced inflammation

Female WT and KO mice were subcutaneously injected in the right hind paw plantar surface with 2% carrageenan λ freshly prepared in saline. 30 μL of saline were injected as control in the controlateral paw. No major adverse effects were observed after injection of 2% carrageenan in the right paw of the mice. All the animals stayed alive until the end of the experiment.

#### Cytometric Beads Array

Peripheral inflammatory responses to CARR were analyzed for six induced cytokines: TNFα, MCP-1, IL-6, IL-10, IFN-γ, IL-12p70, using a CBA kit. CBA analysis was performed on the plasma of control (n = 4 per genotype) and 2% CARR-treated PTPH1-WT and KO female mice. No significant cytokine modulation was detected in healthy and treated WT and KO mice 24 hours after carrageenan injection (data not shown).

#### Paw thickness

**Figure 1:**
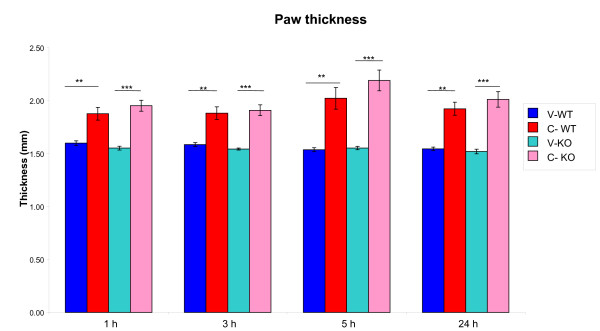
**Carrageenan-induced paw edema in PTPH1-WT and KO mice**. Paw edema was detectable at 1h after treatment in both genotypes at the same intensity. This increased thickness of the paws was maintained till sacrifice, at 24h post CARR-injection. No genotype-related differences were detectable between WT and KO CARR-treated groups. 2way Anova followed by Paired T-test  *:p<0.05; **:p<0.01; ***:p<0.001. V-WT: vehicle-treated PTPH1-WT mice; C-WT: CARR-treated PTPH1-WT mice; V-KO: vehicle-treated PTPH1-KO mice; C-KO: CARR-treated PTPH1-KO mice.

Significantly increased paw thickness was measured by a precision caliper in the CARR-treated paws, compared to the controlateral vehicle treated ones (Figure 1). This increment was statistically significant in both WT and KO groups and was already detectable 1 hour after carrageenan injection. The edema was still present 24 hours post-carrageenan injection (PTPH1-WT: P_1h_=0.0028; P_3h_=0.0022; P_1h_=0.0049; P_1h_=0.0006) (PTPH1-KO: P_1h_=0.0001; P_3h_=0.0002; P_1h_=0.0002; P_1h_=0.0003) (Figure 1). No statistical differences in paw thickness were detected in PTPH1-WT *versus *PTPH1-KO animals.

#### Behavioral Tests

##### Hargreaves's test

**Figure 2:**
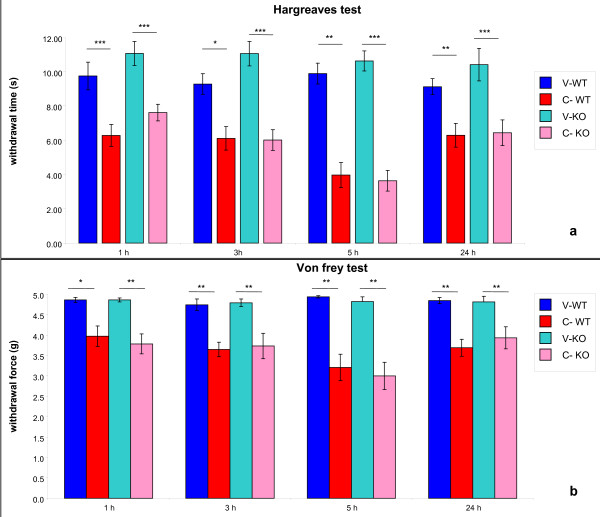
**Behavioral tests performed on CARR-treated PTPH1-WT and KO mice**. **a) **Withdrawal force measured at the Von Frey test was significantly decreased by CARR treatment in both WT and KO mice, starting 1 hour after CARR injection, till 24 hours. The peak was reached 5 hours after CARR treatment. **b) **The withdrawal time measured at Hargreaves' test was significantly decreased by CARR treatment in both WT and KO mice, starting 1 hour after CARR injection, till 24 hours. The peak of response was reached 5 hours after CARR treatment. No genotype-related differences were detectable between WT and KO CARR-treated groups at both tests. 2way Anova followed by Paired T-test *:p < 0.05; **:p < 0.01; ***:p < 0.001. V-WT: vehicle-treated PTPH1-WT mice; C-WT: CARR-treated PTPH1-WT mice; V-KO: vehicle-treated PTPH1-KO mice; C-KO: CARR-treated PTPH1-KO mice.

CARR-treated paws showed a significant decrease in the Hargreaves'test response compared to the controlateral vehicle treated ones (Figure 2a). This reduced withdrawal time was statistically significant in both WT and KO groups; it was detectable already at 1 hour after carrageenan injection through 24 h maintaining the same intensity (PTPH1-WT: P_1h_=0.00004; P_3h_=0.0122; P_1h_=0.0016; P_1h_=0.0039) (PTPH1-KO: P_1h_=0.0001; P_3h_=0.00001; P_1h_=0.0005; P_1h_=0.0005) (Figure 2). No statistical differences in withdrawal time were detected in PTPH1-WT versus PTPH1-KO animals (Figure 2a).

##### Von Frey test

CARR injection also induced a significantly decreased response at the Von Frey test compared to the controlateral vehicle treated paw (Figure 2b). Again, the reduction observed in mice undergoing this test was statistically significant in both PTPH1-WT and KO groups, already detectable at 1 hour after carrageenan injection and maintained through 24 h with the same intensity (PTPH1-WT:P_1h_=0.017; P_3h_=0.0002; P_1h_=0.001621; P_1h_=0.002458) (PTPH1-KO: P_1h_=0.004; P_3h_=0.00977; P_1h_=0.001272; P_1h_=0.007833) (Figure 2b). No statistical differences in withdrawal force were detected between the two genotypes.

##### CatWalk test

##### Print area

**Figure 3:**
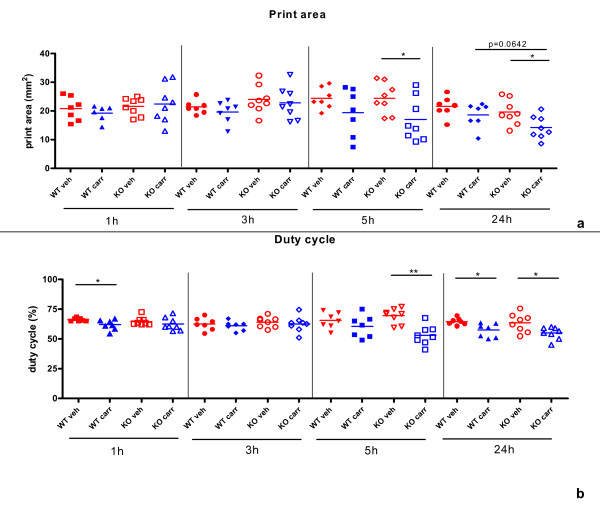
**Catwalk analysis performed on CARR-treated PTPH1-WT and KO mice**. **a) **The print area parameter was not modulated by CARR injection in both genotypes at 1 and 5 hours post-treatment; a slight CARR-induced decrease in print area was detectable in both genotypes at 5 and 24 hours post-treatment and it was statistically significant only in KO mice group, at both time points. A trend in genotype-related difference between CARR-treated animals (WT and KO) was recorded 24 h after challenge. 2way Anova followed by Paired T-test *:p < 0.05; **:p < 0.01; ***:p < 0.001. **b) **The duty cycle parameter was significantly altered by CARR injection in WT mice 1 h post-treatment, but no difference was detected in KO mice group. No CARR-induced or genotype-induced differences in duty cycle were detectable 3 hours after CARR treatment. A slight CARR-induced decrease duty cycle was detectable in WT group at 5 hours post-treatment, while a strong down-regulation of this parameter was recorded in KO group. 24 hours after CARR treatment both genotypes displayed a decrease percentage of duty cycle and no genotype-related differences were detectable between WT and KO CARR-treated groups. 2way Anova followed by Paired T-test *:p < 0.05; **:p < 0.01; ***:p < 0.001.

No differences in print area due to either treatment or genotype were detectable at 1 and 3 hours post CARR-injection. At 5 and 24 hours post-injection, KO CARR-treated paws showed a significant decreased print area compared to controlateral vehicle-treated paws (P_KO5 h _< 0.05; P_KO24 h _< 0.05). No differences were detected in WT CARR-treated *vs *vehicle-treated paws at 5 hours post-injection, but a trend in decreased print area was present in WT CARR-treated *vs *vehicle-treated paws at 24 hours time point (P _WT24 h _= 0.0642) (Figure 3a).

##### Duty cycle

In the WT group, a slight significant decrease in duty cycle was detectable in the CARR-treated paws compared to the vehicle-treated ones, already 1 hour after CARR-injection (P_WT1 h _< 0.05; Figure 3b). At this time point, no significant differences were found within the KO mice group (CARR *vs *vehicle treated animals) nor between WT and KO mice. No differences in duty cycle due either to treatment or to genotype were detectable at 3 hours post CARR-injection. At 5 hours post CARR-injection, PTPH1-KO CARR-treated paws displayed a significant decreased duty cycle compared to the controlateral vehicle-treated one (P_KO5 h _< 0.01), but not compared to the WT CARR-treated animals. This difference was maintained in KO mice, CARR *vs *vehicle, also at 24 hours post-injection (P_KO24 h _< 0.05), and it was detectable also in the WT mice group (CARR *vs *vehicle P_WT24 h _< 0.05) (Figure 3b).

#### Histological Analysis

Vehicle treatment did not induce any signs of inflammation in both PTPH1-WT and KO mice (data not shown).

**Figure 4:**
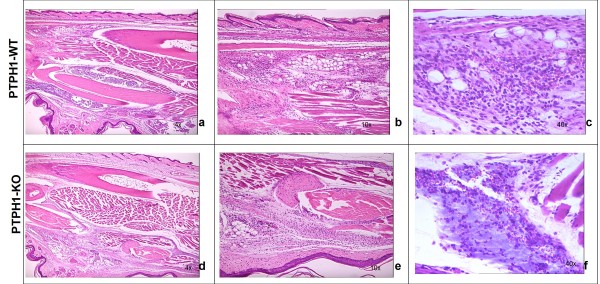
**H&E staining on PTPH1-WT and KO CARR-treated paws**. **a) **PTPH1-WT paws 24 h after CARR treatment displayed a strong inflammation (4×), **b) **severe cellular infiltration (10×) and **c) **also red cells presence, indicating hemorrhage (40×). **d) **PTPH1-KO CARR-treated paws presented the same level of inflammation as matched WT paws (4×), **e) **with a strong presence of immune cells (10×) and **f) **red cells (40×).

Carrageenan treatment induced a moderate to severe acute inflammation in the paws of both PTPH1-WT (Figure 4a-4c) and KO mice (Figure 4d-4f) compared to vehicle treatment 24 hours after challenge. Neutrophil infiltration and hemorrhage, represented by red cell presence, were detected in CARR-treated mice 24 hours after injection (Figure 4c, 4f). No genotype-related differences were noted by simple visual observation in the paw architecture or in cellular infiltration at this late time point.

#### LPS-induced inflammation

Female mice (PTPH1-WT and KO) received an ip injection of 1 mg/kg of LPS and were sacrificed by an ip overdose of thiopental at 30, 60 and 180 minutes after LPS injection. No major side or toxic effects were observed after ip injection of LPS in PTPH1-WT and KO female mice. All the animals stayed alive until the end of the experiment. At sacrifice, blood was collected and plasma and total white cell populations were isolated, as previously described. RT-PCR on white cells was performed for cytokine genes.

#### RT-PCR on cytokine-related genes

RT-PCR was carried out for the following cytokine genes: TNFα, ccl2 (MCP-1), IL12a, IL-1β, IL-6, IL-10 and IL-2.

**Figure 5:**
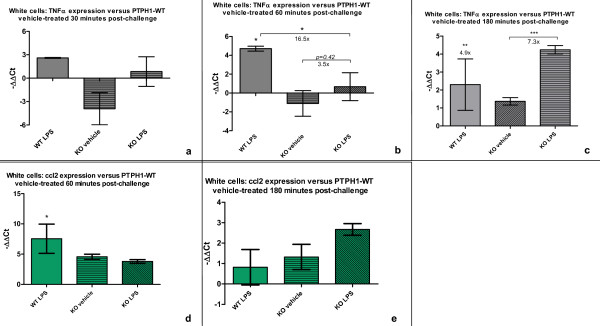
**RT-PCR on white cells of LPS-treated WT and KO mice**. Graphical representation of minus ΔΔCt values of WT LPS-treated, KO vehicle-treated and KO LPS-treated calculated *versus *WT vehicle-treated. **a) **PTPH1- WT and KO mice displayed a trend in LPS-induced increased expression of TNFα in white cells 30 after treatment, that **b) **became significant 60 mpt in WT mice; at this time point also a genotype-related difference in TNFα expression was recorded between WT and KO mice; **c) **at 180 minutes TNFα levels were significantly increased by LPS in both WT and KO mice. **d) **60 minutes post challenge LPS-induced 187 fold increase in ccl2 gene expression in WT white cells was detected, and no difference in KO mice **e) **No difference was detected in ccl2 white cells expression of WT and KO mice, 180 minutes after LPS treatment. 2way Anova followed by T-test; *:p < 0.05; **:p < 0.01; ***:p < 0.001.

TNFα gene expression levels were slightly increased in white cells of LPS-treated mice compared to vehicle-treated animals 30 minutes post treatment (mpt) in both genotypes (Figure 5a).

At 60 mpt, TNFα mRNA levels of PTPH1-WT LPS-treated mice were significantly higher (26 fold) compared to vehicle-treated WTs (P_WT 60 mpt _< 0.05; Figure 5b). At this time point, PTPH1-KO white cells displayed a trend of increased TNFα mRNA levels (3.5 fold) in LPS-treated *vs *vehicle-treated mice (P_KO 60 mpt _= 0.42). Two-way Anova analysis pointed out a genotype-related decrease of TNFα gene expression (16.5 fold) in the LPS-treated mice group, KO *vs *WT (P_KOvsWT _< 0.05; Figure 5b).

At 180 mpt, both PTPH1-WT and KO LPS-treated mice showed a highly significant increase of TNFα expression in whole white cells compared to vehicle-treated animals (4.9 and 7.3 fold respectively) (PTPH1-KO LPS *vs *vehicle; P_KO 180 mpt _< 0.001; PTPH1-WT LPS *vs *vehicle; P_WT 180 mpt _< 0.01; Figure 5c). No genotype-related differences in TNFα mRNA level were recorded at this late time point.

Ccl2/MCP1 gene expression in white cells showed a significant increase (187 fold) in WT LPS-treated compared to vehicle-treated mice at 60 mpt (Figure 5d), whereas no alteration was observed in KO mice or within or between genotypes, at 30 (data not shown) and 180 minutes post treatment (Figure 5e).

IL1β, IL6, IL-10, IL-2 and IL12a expression levels were not significantly altered in total white cells extracted from LPS-treated *vs *vehicle-treated in both PTPH1-WT and KO mice at any time point investigated (data not shown).

#### Cytometric Beads Array

Six cytokines (TNFα, MCP-1, IL-6, IL-10, IFN-γ, IL-12p70) were analyzed in the plasma of LPS- and vehicle-treated WT and KO mice. IFN-γ and IL-12p70 release did not display a significant modulation in our mouse model at the time points investigated. Values recorded below detection limit were excluded from the final analysis.

##### • 30 minutes post treatment

**Figure 6:**
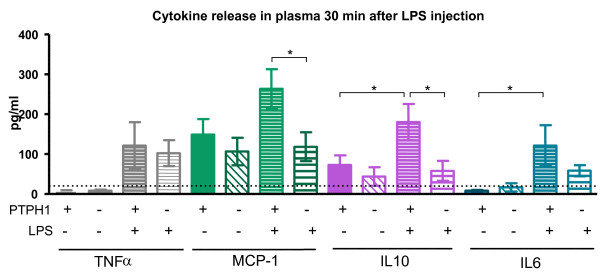
**CBA analysis on plasma 30 minutes after 1 mg/kg LPS injection**. Genotype-related difference in MCP-1 and IL10 between WT and KO LPS-treated animals. WT animals displayed a LPS-induced increase in IL10 and IL6; dot line indicates the detection limit of CBA kit, as reported by the supplier. 2way Anova followed by Bonferroni post-hoc test; *:p < 0.05; **:p < 0.01; ***:p < 0.001.

TNFα overall release in the plasma was increased in LPS-treated mice compared to vehicle-treated ones 30 minutes post LPS injection (P_2way _= 0.0199), but Bonferroni's post hoc test revealed no significant differences in the PTPH1-WT and KO groups associated with either treatment or genotype (Figure 6).

MCP-1 levels were slightly modulated in WT LPS-treated plasma compared to the vehicle-treated group at 30 mpt, while no difference in the KO mice group was detectable at this time point. A genotype-related 50% decrease in MCP-1 release in plasma was recorded in LPS-treated KO *vs *WT mice (P_KOvsWT _< 0.05).

IL10 levels in plasma were significantly increased due to LPS treatment in WT mice at 30 minutes (165%, P_WT _< 0.05), whereas no difference in the KO mice group was found. However, a genotype-related decrease in IL10 release in plasma was detectable in LPS-treated KO *vs *WT mice (150%, P_KOvsWT _< 0.05).

IL6 release was significantly higher in the plasma of WT LPS-treated compared to vehicle-treated mice 30 minutes post LPS injection (P_WT _< 0.05), while no modulation in IL6 levels was seen in KO mice, LPS vs vehicle. No genotype-related differences in IL6 release were detected in LPS- treated WT vs KO animals.

##### • 60 minutes post treatment

**Figure 7:**
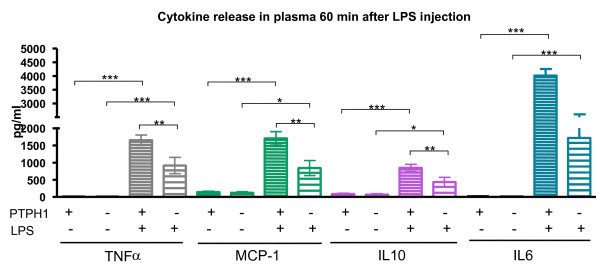
**CBA analysis on plasma 60 minutes after 1 mg/kg LPS injection**. Genotype-related difference detected in TNFα, MCP-1 and IL10 between WT and KO LPS-treated animals. Both WT and KO mice displayed a LPS-induced increase in TNFα, MCP-1 and IL10; IL6 level was significantly increased by LPS in both WT and KO mice. 2way Anova followed by Bonferroni post-hoc test; *:p < 0.05; **:p < 0.01; ***:p < 0.001.

At 60 minutes post LPS/vehicle injection TNFα, MCP-1, IL-6 and IL-10 release was highly significantly increased in the plasma of LPS-treated animals compared to vehicle-treated mice in both WT and KO groups (Figure 7). Moreover, a genotype-related 50% decrease (P_KOvsWT _< 0.01) in TNFα, MCP-1 and IL-10 plasma level was seen in LPS-treated KO *vs *WT mice (Figure 7). No genotype-related differences were detected in IL-6 plasma release between PTPH1-WT and KO animals (Figure 7).

##### • 180 minutes post treatment

**Figure 8:**
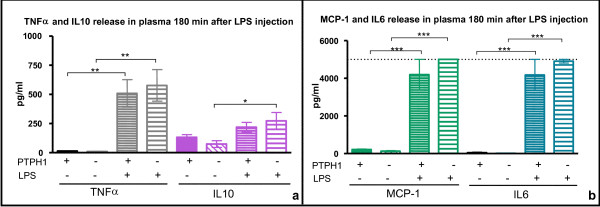
**CBA analysis on plasma 180 minutes after 1 mg/kg LPS injection**. **a) **PTPH1- WT and KO mice displayed a LPS-induced increase in TNFα and IL10; **b) **MCP-1 and IL6 levels were significantly increased by LPS in both WT and KO mice; dot line indicates the detection limit of CBA kit, as reported by the supplier. 2way Anova followed by Bonferroni post-hoc test; *:p < 0.05; **:p < 0.01; ***:p < 0.001.

At 180 minutes post LPS/vehicle challenge, TNFα (Figure 8a), MCP-1 and IL-6 (Figure 8b) levels were significant increased in the plasma of WT and KO LPS-treated compared to vehicle-treated mice, but no genotype-related differences were detectable. IL10 release was significantly increased in PTPH1-KO LPS-treated mice *vs *their vehicle controls (Figure 8a) (P_KO _< 0.05), while no modulation was recorded in WT mice group. No genotype-related effect on IL10 release was detectable between WT and KO mice.

## Discussion

PTPH1 has been proposed to act as a negative TCR regulator *in vitro*, interacting and dephosphorylating the TCRζ chain [13-15] but these results have not been confirmed by *ex vivo *studies on primary PTPH1-KO T cells [16,17]. Therefore, we sought to ascertain whether PTPH1 could have an effect on immune system upon inflammatory challenge, thus in the complex *in vivo *machinery. Two inflammatory mouse models were used to test the impact of PTPH1 deletion on the immune system: carrageenan- and LPS-induced inflammation.

Carrageenan λ is a sulfated polysaccharide derived from red seaweed that is able to activate the innate immune response. CARR interacts with TLR4 leading to increased Bcl10, to NFκB pathway activation and IL8 production [25,26]. CARR injection in the hind paw of the mouse is one of the most commonly used models of inflammation and inflammatory pain and it has a biphasic profile [27]. Recent studies pointed out important roles for prostaglandins, nitric oxide and TNFα in the CARR-induced inflammatory response [27-29]. In particular, it has been shown that TNFα is involved in both phases of mouse carrageenan-induced edema. Thus, TNFα has a strong relevance not only in inflammatory events, but also on nociceptive response and on neutrophil migration induced by carrageenan in mice [29]. Soluble TNFα is processed from its pro-protein form by a specific sheddase, called TACE [30,31], that is also responsible for the processing of other cytokines and cytokine receptors [32-35]. Interestingly, PTPH1 is known to inhibit TACE expression and activity *in vitro *[36]. We therefore analyzed cytokines plasma levels in carrageenan-treated WT and KO mice, but no variation was found between genotypes using this inflammatory agent (data not shown), in agreement with a previous CBA study on the rat carrageenan model [37]. We conclude that local 2% carrageenan stimulation might not be sufficiently potent to unmask a phenotype in cytokine modulation in PTPH1-KO mice at plasma level, and that hind paw and muscle cytokine concentrations should be analyzed in both genotypes, to unravel PTPH1 role in local cytokine release.

As already mentioned, the CARR-induced model has a biphasic profile, that is characterized by an early development of edema, that peaks at 6 h and then again at 72 h [27]. In the present study, carrageenan injection induced paw edema in both PTPH1-WT and KO mice, detectable already 1 hour after injection and persistent till 24 h (Figure 1), showing no differences in intensity between the genotypes. Furthermore, carrageenan challenge induced a marked neuthrophils migration to the site of injection 24 h after treatment (Figure 4) [27]. Another hallmark of carrageenan stimulation is a long-lasting reduction in the threshold to nociceptive stimuli, that was evident in our model already 1 h after challenge and was sustained for up to 72 h [27,38,39]. PTPH1-KO mice did not show any significant difference in neutrophils infiltration (Figure 4) or in pain behavior both at Von Frey's and Hargreaves' tests, compared to WT littermates (Figure 2a, 2b). These findings suggest that PTPH1 does not play a major role in the inflammatory-induced transmission and integration of the allodynic and painful stimuli. Comparatively, Catwalk gait analysis showed a trend of slightly earlier onset (5 h after injection) of spontaneous pain perception indicated as print area (Figure 3a) and duty cycle (Figure 3b) in PTPH1-KO mice, compared to matched WTs. Pilecka and colleagues recently showed that PTPH1 is expressed also in skeletal muscles [40]. Despite no differences were detected in grip strength test between PTPH1-WT and KO mice in basal condition (data not shown), Catwalk data might also suggest a possible role of PTPH1 in muscle fatigue. Further tests should be performed on PTPH1-WT and KO mice upon challenge, in order to unravel the underlying molecular mechanisms.

Carrageenan and LPS challenges are frequently used in rodents as models to investigate innate immune response mechanisms [41-43]. LPS is a major component of the outer membrane of Gram-negative bacteria and it is a critical player in the pathogenesis of septic shock [44]. Like carrageenan, LPS binds to the MD2-TLR4 complex and activates both MyD88-dependent and independent (TRIF-dependent-TIR-domain-containing adapter-inducing interferon-β) pathways [45-47]. The MyD88-dependent pathway results in the activation of TRAF6 (TNF Receptor Associated Factor 6) and in the immediate activation of NFκ B, MAPK and JNK pathways, leading to the early production of pro-inflammatory cytokines as TNFα, IL-1β, IL6 and MCP-1 [48-50]. MyD88-independent pathway results in rapid activation of the interferon regulatory factors (IRF) 3 and 7 that induces the production of IFNβ and consequently IFNα, nitric oxide production and delayed NFκB activation [48,50], leading to late cytokine production. LPS challenge on PTPH1-KO and WT mice aimed to understand the possible role of this phosphatase in the inflammatory process and in particular in cytokine expression and release. Thus, CBA analysis was performed on the plasma of LPS- and vehicle-treated WT and KO mice (IL-10, IL-12p70, TNFα, IL-6, MCP-1, IFN-γ).

IL10 is an immunomodulatory cytokine, whose production is rapidly induced by monocyte/macrophages upon LPS challenge [51]. IL10 treatment *in vitro *is known to negatively regulate LPS responses [51], in particular inhibiting the induction of pro-inflammatory cytokines, as TNFα, IL12 [52,53], IL-1α, and IL-6 [54,55]. Both PTPH1-WT and KO mice displayed significantly increased IL10 plasma level upon LPS challenge, but no significant reductions of IL-6, TNFα and thus MCP-1 plasma levels were detected in LPS-treated *vs *vehicle-treated PTPH1-WT and KO mice. Interestingly, IL10 levels were reduced in LPS-treated KO mice plasma compared to WTs, at 30 and 60 minutes after challenge (Figure 6, 7); no increased MCP-1, TNFα, and IL6 plasma levels were detected in LPS-treated KO *vs *WT mice at these time points. Indeed, IL-10 has not an exclusive pro-inflammatory action [56] and it has been demonstrated in an LPS-model that IL-10 release increases as MCP-1, IL-6 and TNFα [57]. Thus, our results on overall increased cytokines levels upon LPS treatment are in accordance with these studies. Comparatively, an overall decrease in cytokines release was recorded at 30 and 60 minutes post challenge in LPS-treated PTPH1-KO *vs *WT mice (Figure 6, 7).

Several studies reported that IL10 up-regulates the expression of socs1 and socs3 genes, blocking the IFNs-induced JAK/STAT pathway [51,58] and stimulating the expression of PTP1B [51]. It has recently been demonstrated that the production of IL10 by human Treg cells is enhanced by IL2 signaling via activation of STAT5 molecules [59,60]. PTPH1 is known to dephosphorylate STAT5b *in vitro *[61], and therefore the IL10 reduction in PTPH1-KO plasma after 30 minutes and 1 hour post LPS injection appears counterintuitive (Figure 6, 7). These data could indicate PTPH1 as a possible target of the early MyD88-dependent pathway, which acts on the overall pro- and anti-inflammatory cytokines production, but further investigations are needed to support this hypothesis and to identify PTPH1 substrates.

LPS-induced IL6 release was detectable in WT mice, LPS-treated *vs *vehicle-treated group, as reported by several studies [62,63]. Increased IL6 plasma level of LPS *vs *vehicle-treated mice were recorded also in KO mice, at the three time points investigated. It has been recently shown that JAK2 and STAT5 are required for LPS-induced IL-6 production [63] and, as already mentioned, STAT5b is known as PTPH1 substrate [61]. Thus, the trend in decreased IL6 plasma levels of KO *vs *WT LPS treated mice 60 minutes after challenge could be due to a partial and temporally-limited inactivation of JAK/STAT pathway by PTPH1 deletion. Further biochemical analysis are necessary to confirm this hypothesis and to understand the possible PTPH1 role in this pathway.

It has been widely demonstrated that endotoxin injection leads to a rapid and dose-dependent TNFα expression and release in mice [44,64,65]. The present study demonstrates that LPS injection induced an increased level of TNFα mRNA expression in LPS-treated *vs *vehicle-treated mice in both PTPH1-WT and KO (Figure 5a, 5b, 5c), that is genotypically different only at 60 minutes after endotoxin injection (Figure 5b). At this time point, PTPH1-KO LPS-treated mice displayed lower TNFα expression compared to matched WTs, that was detectable also at the level of TNFα release in plasma (Figure 7). In particular, endotoxin injection led to a rapid and very significant increase of TNFα release in both PTPH1-WT and KO mice at the three time points investigated (Figure 7, 8). In accordance to mRNA level (Figure 5b), TNFα release was significantly lower in LPS-treated KO *vs *matched WT mice 60 minutes post challenge (Figure 7), corroborating the hypothesis of a weaker inflammatory response of PTPH1-lacking mice.

Furthermore, increased MCP-1 plasma levels were recorded in both PTPH1-WT and KO LPS-treated *vs *vehicle-treated mice (Figure 6, 7, 8). TNFα is known to promote ccl2 gene expression [66,67], activating NFκB-inducing kinases, including IκB kinases (IKK). IKKs promote NFκB heterodimers translocation into the nucleus, leading to the transcription of targeted genes [66,67]. Several studies show that both NFκB and MAPK pathways are required for ccl2 induction; specifically, transcription factor AP-1 contributes to TNFα-inducible expression of MCP-1 gene [67-69]. This regulatory mechanism might explain the slight reduction of TNFα and MCP-1 gene expression and release in KO mice 1 hour post challenge (Table 1), when LPS induced the peak of TNFα plasma levels. Indeed, LPS challenge failed to induce a full response, as measured by TNFα expression and release in PTPH1-KO mice, leading to a lower stimulation of MCP-1 and possibly to a consequently reduced monocytes and macrophages recruitment 1 hour after injection. Cytokine levels in PTPH1-KO plasma were comparable to WT 3 hours after LPS injection (Figure 8), suggesting that PTPH1 activity was temporally limited at the peak of TNFα release, or that some compensatory mechanisms might have intervened later on, in the inflammatory process.

**Table 1:** Cytokine expression and release in LPS-treated mice- Summary

LPS-treated KO *vs *WT						
	**Minutes post-injection**					
	
	**30**		**60**		**180**	

**Cytokines**	**Gene**	**protein**	**gene**	**protein**	**gene**	**protein**

**TNFα**	=	=	-	-	=	=
**MCP-1**	=	-	- (ns)	-	=	=
**IL6**	=	- (ns)	=	- (ns)	=	=
**IL12p70**	=	-	=	=	=	=
**IL 10**	nn	-	nn	-	nn	=

Summary of the CBA and RT-PCR results in LPS-treated PTPH1-WT and KO mice at different time points (30, 60 and 180 minutes post- injection). **= **indicates no variation between WT and KO LPS-treated mice; **- **indicates a lower expression/release in LPS-treated KO compared to WT matched ones; - **(ns) **indicates a trend in lower expression/release in LPS-treated KO compared to WT matched ones; **nn **indicates non-performed.

Despite the fact that most pro-inflammatory cytokines are transcribed after NFκB activation [68,70-72], the overall control of production for several cytokines is more complex, and includes other pathways as JAK/STAT, MAPK [73,74], JNK [75,76] and also post-transcriptional and post-translational regulatory steps within these pathways, that could be cytokine-specific. PTPH1 could act at one or more steps of this composite regulatory process, by dephosphorylating key molecules, as indicated by the lack of direct correlation between cytokines expression and release, in particular for ccl2 (Table 1). Proteolytic conversion of pro-proteins into mature cytokines is a further level of control for cytokine production. TACE is involved in the ectodomain release of several cytokines, in particular of membrane-bound TNFα [30,77,78]. As already mentioned, PTPH1 has been reported to be an inhibitor of TACE expression and activity *in vitro *[36], but the mechanism of this inhibition is still unclear.

## Conclusion

In summary, PTPH1-KO mice exhibit a trend of slight increased spontaneous nociceptive perception in the CARR-induced inflammatory pain model. A slight decrease in TNFα expression and a significant overall delayed cytokine release are detectable in PTPH1-KO mice in the early phases of LPS-induced inflammation. In conclusion, the present study highlights a slight potential role for PTPH1 in spontaneous pain sensitivity and more strikingly, it indicates that this phosphatase might play a role in the positive regulation of the LPS-induced cytokines release *in vivo*, in contrast to previous reports indicating PTPH1 as potential negative regulator of immune response [13-15].

## Competing interests

The present work is part of CP's PhD program at the University of Eastern Piedmont, in close collaboration with MerckSerono International S.A VM and PFZ are former employees of MerckSerono International S.A RH and BG are employed by MerckSerono International S.A. which is involved in the discovery and the commercialization of therapeutics for the prevention and treatment of human diseases.

## Authors' contributions

The study was devised by CP and VM and carried out by CP. DL performed the behavioral tests (Von Frey, Hargreaves and Catwalk) on PTPH1-WT and KO animals. VM, BG and RH have been deeply involved in the first editing of the manuscript and all the authors contributed to modifications in subsequent drafts. PFZ and RH have given the final approval of the version to be published. All the authors read and approved the final version of the manuscript.
